# Supplementary material for: Caenorhabditis elegans Uses Canonical and Non-canonical Hippo signaling
Source: bioRxiv. 2025 Aug 29:2025.08.22.671798. Preprint. [Version 3] doi: 10.1101/2025.08.22.671798 (PMC12407797; doi:10.1101/2025.08.22.671798)
Supplement: Supplement 1 [file NIHPP2025.08.22.671798v3-supplement-1.pdf]

Huynh Figure S1

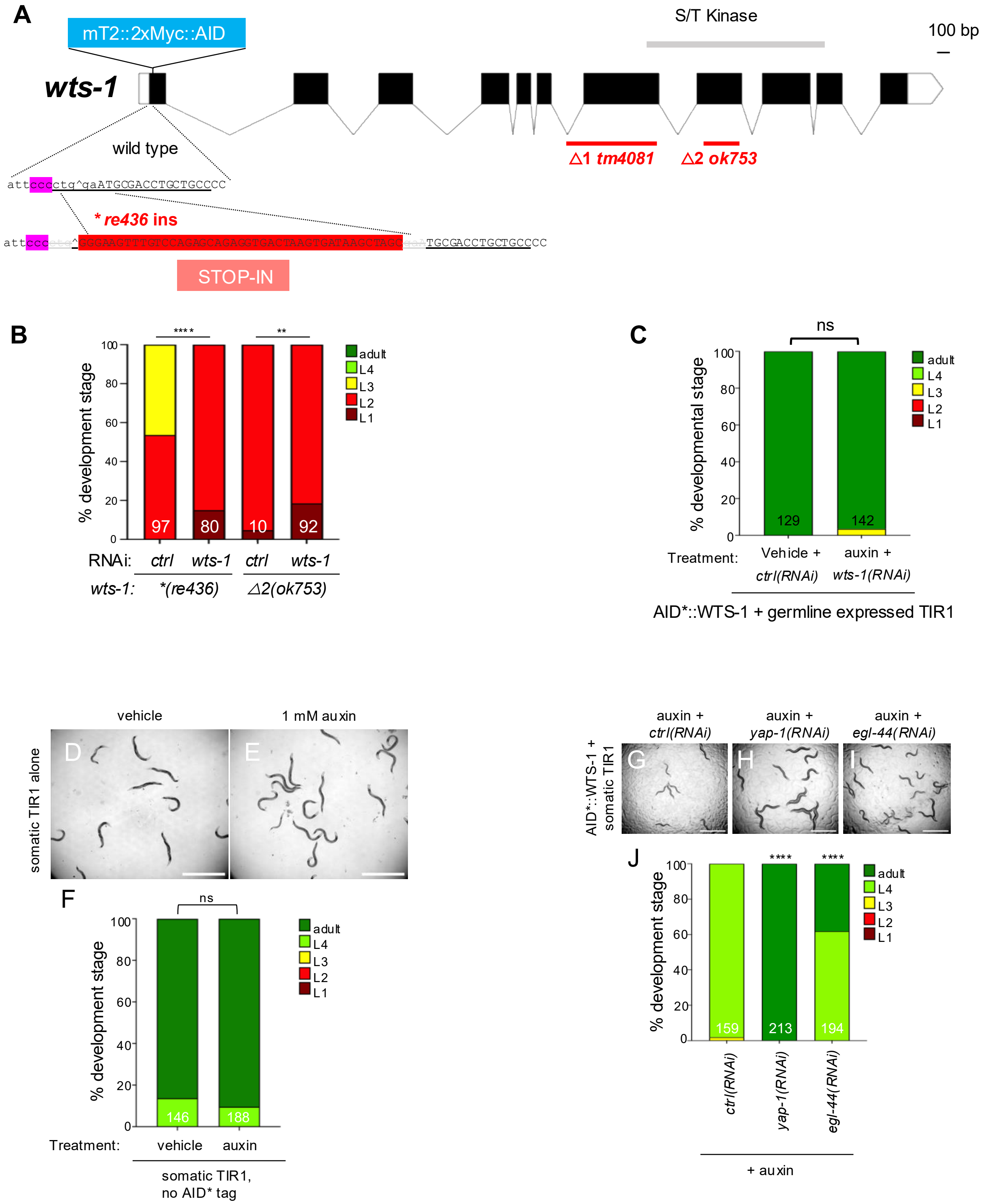

Huynh Figure S2

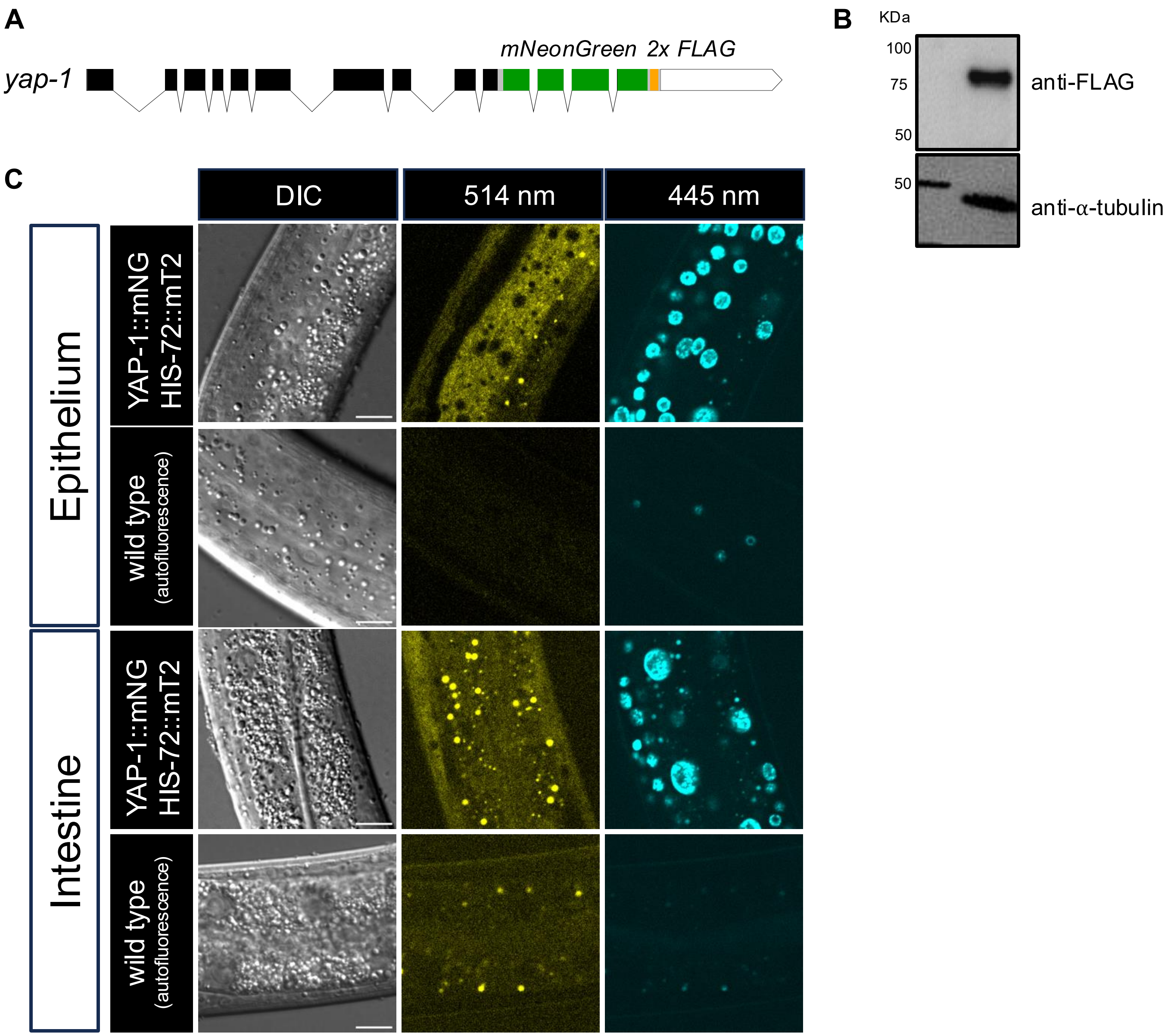

Huynh Figure S2, continued

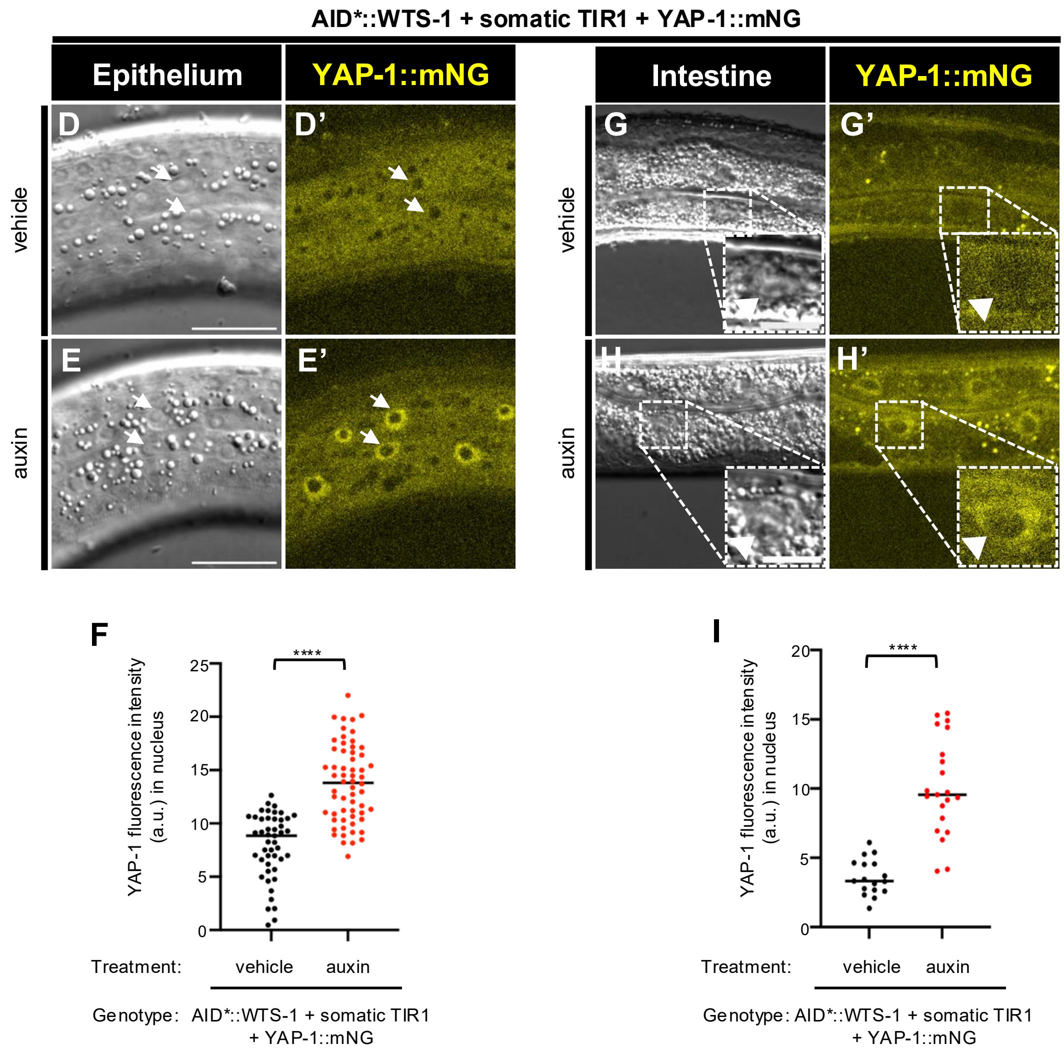

Huynh Figure S2, continued 2

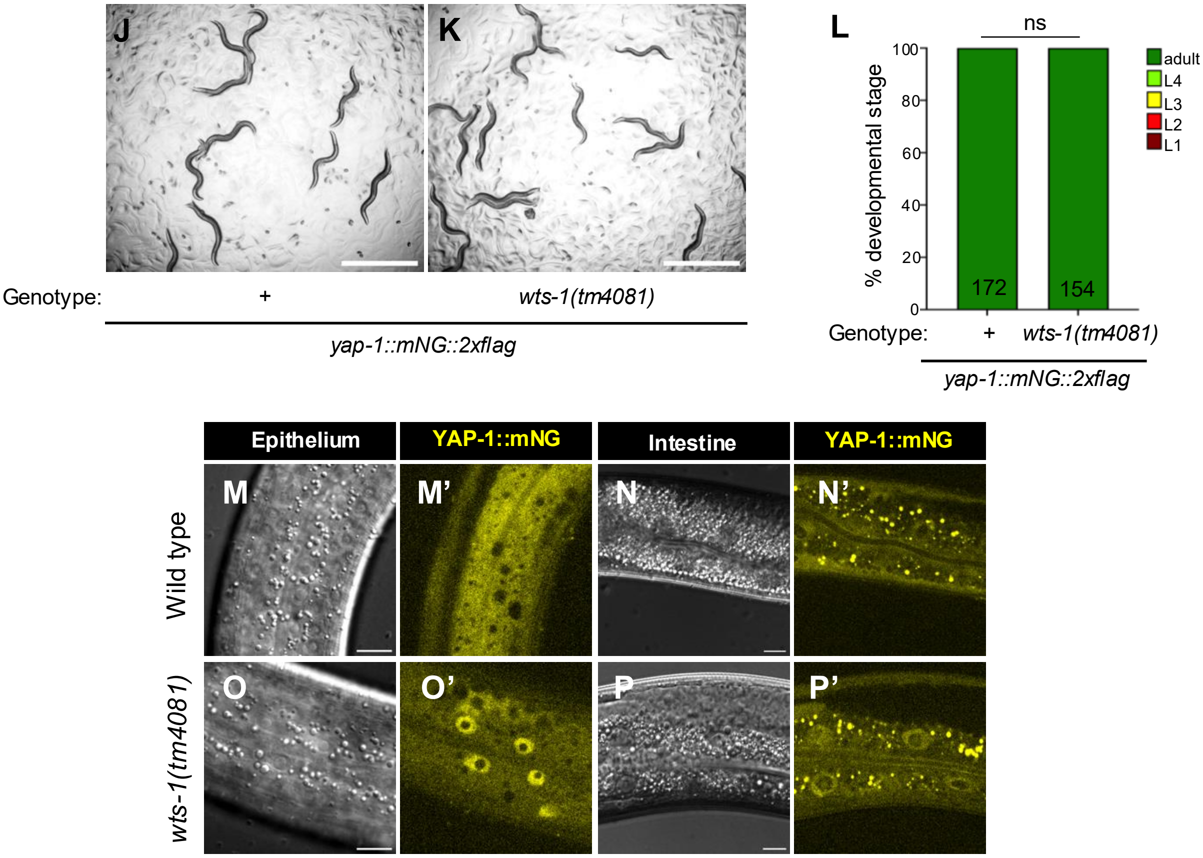

Huynh Fig. S3

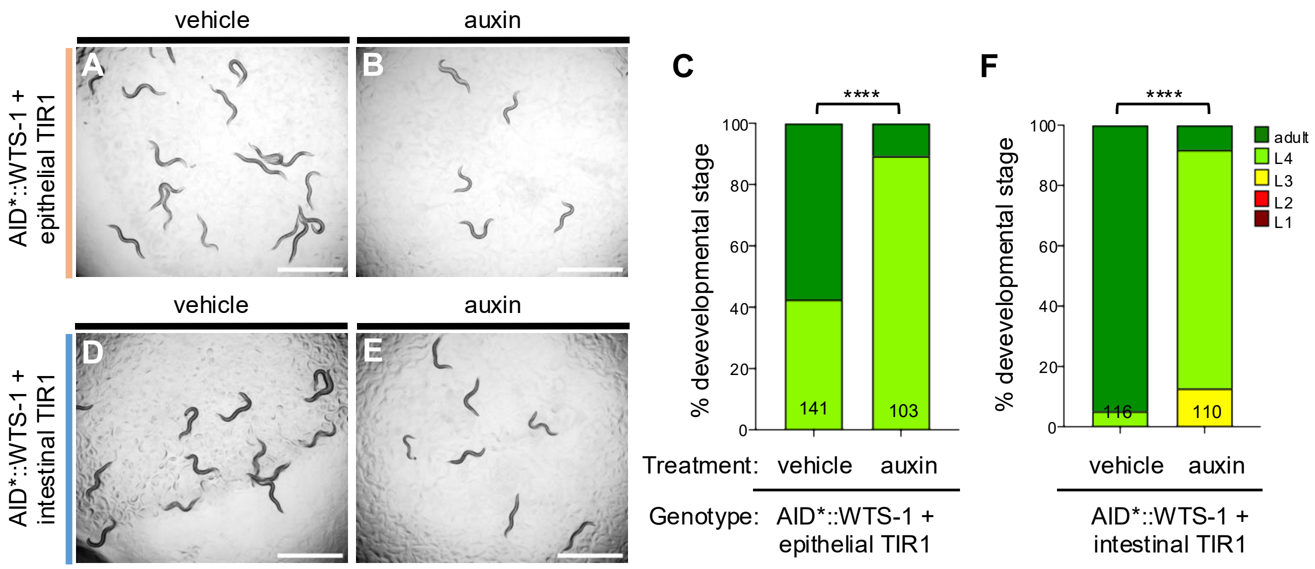

vehicle
auxin
A
B
AID*::WTS-1 +
epithelial TIR1
vehicle
auxin
D
E
AID*::WTS-1 +
intestinal TIR1
C
F
****
****
% devevelopmental stage
100
80
60
40
20
0
141
103
116
110
adult
L4
L3
L2
L1
Treatment: vehicle auxin
Genotype: AID*::WTS-1 + epithelial TIR1
vehicle auxin
AID*::WTS-1 + intestinal TIR1

bioRxiv preprint doi: https://doi.org/10.1101/2025.08.22.671798; this version posted August 29, 2025. The copyright holder for this preprint (which was not certified by peer review) is the author/funder, who has granted bioRxiv a license to display the preprint in perpetuity. It is made available under aCC-BY 4.0 International license.

Huynh Fig S4A

```
Lt blue: Ste20 S/T kinase
Yellow: SARAH coiled coil


CLUSTAL O(1.2.4) multiple sequence alignment

CeCST-1      --MPPST-----DSSRRNSEEGSSDGFKLDSSALNKPPEEVFDIVGKLGEGSYGSVHKAI   53
DmHpo        MSEPEVTSVVDMKSPNISSSCSFFKLKKLSEESLLQPPEKVFDIMYKLGEGSYGSVYKAV   60
HsMST1/STK3  -----------ME----QPPAPKSKLKKLSEDSLTKQPEEVFDVLEKLGEGSYGSVFKAI   45
HsMst2/STK4  --------METVQ----LRNPPRRQLKKLDEDSLTKQPEEVFDVLEKLGEGSYGSVYKAI   48
                         .           .  **...:* : **:***:: **********.**:

CeCST-1      HRESGHVLAIKKVPVDTDLQEIIKEISIMQQCKSKYVVKYYGSYFKHSDLWIVMEYCGAG   113
DmHpo        HKESSSIVAIKLVPVESDLHEIIKEISIMQQCDSPYVVRYYGSYFKQYDLWICMEYCGAG   120
HsMST1/STK3  HKESGQVVAIKQVPVESDLQEIIKEISIMQQCDSPYVVKYYGSYFKNTDLWIVMEYCGAG   105
HsMst2/STK4  HKETGQIVAIKQVPVESDLQEIIKEISIMQQCDSPHVVKYYGSYFKNTDLWIVMEYCGAG   108
             *:*:. ::*** ***::**:************.* :**:*******: **** *******

CeCST-1      SISDIMRARRKPLSEQEISAVLRDTLKGLQYLHDLKKIHRDIKAGNILLNTDGIAKLADF   173
DmHpo        SVSDIMRLRKKTLTEDEIATILSDTLQGLVYLHLRRKIHRDIKAANILLNTEGYAKLADF   180
HsMST1/STK3  SVSDIIRLRNKTLIEDEIATILKSTLKGLEYLHFMRKIHRDIKAGNILLNTEGHAKLADF   165
HsMst2/STK4  SVSDIIRLRNKTLTEDEIATILQSTLKGLEYLHFMRKIHRDIKAGNILLNTEGHAKLADF   168
             *:***:* *.* * *:**:::* .**:** ***  :********.******:* ******

CeCST-1      GVAGQLTDTMAKRNTVIGTPFWMAPEVIEEIGYDTKADIWSLGITAIEMAEGRPPYSDIH   233
DmHpo        GVAGQLTDTMAKRNTVIGTPFWMAPEVIEEIGYDCVADIWSLGITALEMAEGKPPYGEIH   240
HsMST1/STK3  GVAGQLTDTMAKRNTVIGTPFWMAPEVIQEIGYNCVADIWSLGITSIEMAEGKPPYADIH   225
HsMst2/STK4  GVAGQLTDTMAKRNTVIGTPFWMAPEVIQEIGYNCVADIWSLGITAIEMAEGKPPYADIH   228
             ****************************:****:  *********::*****:***.:**

CeCST-1      PMRAIFMIPTKPPPTFKKPEEWSSEFNDFIRSCLIKKPEERKTALRLCEHTFIKNAPGCD   293
DmHpo        PMRAIFMIPQKPPPSFREPDRWSTEFIDFVSKCLVKEPDDRATATELLEHEFIRNAKHRS   300
HsMST1/STK3  PMRAIFMIPTNPPPTFRKPELWSDDFTDFVKKCLVKNPEQRATATQLLQHPFIKNAKPVS   285
HsMst2/STK4  PMRAIFMIPTNPPPTFRKPELWSDNFTDFVKQCLVKSPEQRATATQLLQHPFVRSAKGVS   288
             ********* :***:*::*: ** :* **: .**:*.*::* ** .* :* *::.*   .

CeCST-1      IMQLMIQDAQEKAILGQAPMA------AS-----------SGNDATLL----SEGMSTMI   332
DmHpo        ILKPMLEETCAIREQQRANRSFGGVLAASQAKSLAT--QENGMQQHITDNAFMEDPGTLV   358
HsMST1/STK3  ILRDLITEAMEIKAKRHEEQQ----------RELEE-EEENSDEDEL-------DSHTMV   327
HsMst2/STK4  ILRDLINEAMDVKLKRQESQQ----------REVDQDDEENSEEDEM-------DSGTMV   331
             *:: :: ::       :                       .. :  :       .  *::

CeCST-1      D--GG--------ESTLVQHKDNYVTA-----QSLR-----------------SQME---   357
DmHpo        PEKFGEYQQSSASDATMIAHAEQGVDEGTLGPGGLRNLSKAAAPAAASSAASPLDMPAVD   418
HsMST1/STK3  KTSV--------------------ESV-----GTMRATSTM------SEGA--QTMI---   351
HsMst2/STK4  RAVG--------------------DEM-----GTVRVASTM------TDGA--NTMI---   355
                                               :*                   *

CeCST-1      -------SLRIGGEIPKS-AYSSSKNGNSPRVQPPGHTASASDPSKNQPFAQ----DGTG   405
DmHpo        SGTMVELESNLGSTMVINSDSDDSTTAKNNDDQKPR---NRYRPQFLEHFDRKNAGDGRG   475
HsMST1/STK3  EHNSTMLESDLG-TMVINSEDEEEEDGTMKRNATSP---QVQRPSFMDYFDKQDFKNKSH   407
HsMst2/STK4  EHDDT-LPSQLG-TMVINAEDEE-EEGTMKRRDETM---QPAKPSFLEYFEQKEKENQIN   409
                       :*  :  .   ..   ..           .   *.  : * :    :
```

bioRxiv preprint doi: https://doi.org/10.1101/2025.08.22.671798; this version posted August 29, 2025. The copyright holder for this preprint (which was not certified by peer review) is the author/funder, who has granted bioRxiv a license to display the preprint in perpetuity. It is made available under aCC-BY 4.0 International license.

```
CeCST-1      PNF-------------------------------QLGTSE-----S----SY--------   417
DmHpo        DEKPIATEYSPAAAEQQQQQQQQQQQQQQDEQHLASGANDLNNWEHNMEMQFQQISAINQ   535
HsMST1/STK3  E-----------------------------------------NCNQNM------------   414
HsMst2/STK4  S------------------------------------------FGKSV------------   415

CeCST-1      -----------KDASYNMMNTE--------------------------------A----E   430
DmHpo        YGLQQHQQQQQVLMAYPLMNEQLIALNNQPNLLLSNAAPMGQQGIPAAAPAQPPPAYQNQ   595
HsMST1/STK3  ------------HEPF----------------------PMS-----------------KN   423
HsMst2/STK4  ------------PGP--------------------------------------------L   419

CeCST-1      YENRFQRAVVDGDFEFLRNITLDELIRRKESLDSEMEEEIRELQRRYKTKRQPILDVIEI   490
DmHpo        HMHTQSHAYVEGEFEFLKFLTFDDLNQRLCNIDHEMELEIEQLNKKYNAKRQPIVDAMNA   655
HsMST1/STK3  VFPDNWKVPQDGDFDFLKNLSLEELQMRLKALDPMMEREIEELRQRYTAKRQPILDAMDA   483
HsMst2/STK4  KNSSDWKIPQDGDYEFLKSWTVEDLQKRLLALDPMMEQEIEEIRQKYQSKRQPILDAIEA   479
                   :   :*:::**:  :.::*  *   :*  ** **.::.::* :*****:*.::

CeCST-1      KKRLQ----------   495
DmHpo        KRKRQQNINNNLIKI   670
HsMST1/STK3  KKRRQQN--------   490
HsMst2/STK4  KKRRQQNF-------   487
             *:: *
```

bioRxiv preprint doi: https://doi.org/10.1101/2025.08.22.671798; this version posted August 29, 2025. The copyright holder for this preprint (which was not certified by peer review) is the author/funder, who has granted bioRxiv a license to display the preprint in perpetuity. It is made available under aCC-BY 4.0 International license.

Huynh Fig. S4B

***cst-1* genomic seq annot:**
Lt gray: UTR
Yellow: exons
Green: alternating exons
Blue: two-codon alternative splice in intron 8.
Purple: exon 9 sequence that is IDENTICAL with *cst-2* exon 9.

>Unspliced + UTR + upstream + downstream (8069bp)
attttcaaaatatatacacccataatatgaaaaagtattttcaactatcaataaacac
cgagaaaagcggaatatcaaaatttttatgaattaggggtgggaggcaattgttaaaatg
cggaaaatattgaattgctgatgaattcaagattcggcgattcccgaaaataccgaatat
tttcaaactcggcaatctggaatagcagacatttagtagggaaacgttgtatgtcttccc
aaaaatatttattacaatgaactattgtttgtttgtttgtttttgaattttatttgttca
aaattatcgattgtcaaattgccaaaaaattttgatattccgcttttctcggtgtttatt
gatagttgaaaatactttttcatattatgggtgtatatatttttgaaaaatcaattaaca
gttctttaaagttaaatagattgaaattaaagaataacttggaaattgtttttgaattta
tgacatctctttgcttgaaaaaatggatgaaacatgctttataaaatcaactagaataat
tgtccaaactgacaaaaagcaatcaactactatgcaccataacaaataaagtctactgac
aagtaaagatagtaaacatacgtgaatcaggtgatgactacccaacaactctcataataa
aacagtaccgcaaaacagcaccaggaaggacacagttagcaatacgatattccgcattca
aacatcctgacgtgtgaagggtcaagtcttatgaaacgagcgcagtacacacttcgcact
ctttcccgtccttttccccctttcgcctcgaccacacactgctcaaataacgtagagtga
gccgagggcattacgaatgctcgtcgaatgtagcgcctgcggtcggttatattaacttct
ttttttttatcacggtacaacttatctgaaatagagtactgtttttgagttgttctctgg
tctttgatagtttgatagtttcaatgggaatatttaattctcacactttcaaaaaggata
tttcaatcttaaatgaaatgctaacattgcgaaatagaattgaaaatcatgatacttcaa
tctctttcatgaaaaataaggttcttctaaatttgtattgttttaaaatatgctcaggtc
tatctcaaagatttcataaattataaaatttattaattagtttttctcggctttttcaac
cattttaccaatgaaatcttaatctaaaaacgttggactttgtcggattactttttttc
caaaagacaattgccgtagtcaattattcattcttcgggccatcggggccacatgggtgg
aaccccgcagacgtcttttcgctttcaaaacgacgtccaaatcgattcgcctgtctttct
cttctcactcttctcgcctcccttcagaatatgaccatacatgcactgttgacgttctaa
ttgaacaagcagagtgcatgtgcaaccaagagaacgcagagaaacatgcgactaatgtgt
cctcaaggataggttttatcttcttcttcttctttcttcttctttcttcgattttcttgc
cccatagttctcgtcggtcggacggacaacatctgctcttcatcttacttatatctcatt
gggtgttcattcctatactcacacaagatcttacaacactttttataaatccttcaact
tgtacacagttaaaggtggagtagcgcaagttggaaaattgtttaaaactactcttatgg
tgtcaaaatgactaaatattaaacaaataaaaatgctgaacagtttttgaaaggacgact
ttcaaaaagtccaacaattggcaaaaactgagtttattttcaattttcaaaaaaaaatca
gataaaatttagaacatctttcagaaactttttgctaaaatatttgattattgtggtgcc
gcatgcatgtttttgagcaatttcagcactgacgttactccacttttaaatttcagcaaa
gttaatttcattaatttcagcgtcctcccg**ATG**CCACCGTCTACAGACAGCTCGCGGCGG
AATTCGGAAGAGGGCTCAAGgtttgtcagacacaaaacaaaaaaaaagagttactcttcc
aaaataaacattctcttttgttttagCGATGGATTTAAATTGGATTCGTCGGCGCTTAAC
AAACCACCAGAAGAAGTTTTCGACATTGTTGGAAAGCTGGGCGAAGGgtttgttatttca
attctatcggattgaaaattccaaattttcagATCATATGGAAGCGTACATAAGGCAAT
TCACAGAGAGTCAGGTCATGTGTTAGCCATCAAAAAAGTGCCAGTGGATACTGATCTTCA

bioRxiv preprint doi: https://doi.org/10.1101/2025.08.22.671798; this version posted August 29, 2025. The copyright holder for this preprint (which was not certified by peer review) is the author/funder, who has granted bioRxiv a license to display the preprint in perpetuity. It is made available under aCC-BY 4.0 International license.

AGAAATTATCAAGGAAATCAGTATAATGCAACAATGTAAAAGTAAATATGTTGTCAAATA
CTATGGATCGTATTTCAAACATTCGGATCTATGGgtgagattgtttagataaattttaag
attttaatgctgtgcccaagtgggactaaataaaagaaattgaacaaaaattatattcgt
tttgtgaattagagttaaaaacagtaaagttctgatttttccacagaaaaaaatgtagct
taaagtggaaataacattcaaaagtattcattcaaatgcatgccaattgaaattcaaaga
ttttattccctacccctagtcgaaaatcaagaagaaaactggactaacaaaaaaactaat
ttaaagATTGTGATGGAATACTGTGGAGCTGGGTCGATCTCCGATATAATGCGTGCCAGG
CGGAAACCGTTATCCGAACAAGAAATCAGTGCAGTTTTGCGCGATACTCTCAAGGGATTG
CAATATTTGCATGACTTGAAGAAGATTCATAGAGATATAAAGgttagaaactgttttttt
ttgaaaaaaaaactgtattttcagGCTGGAAACATTCTACTCAATACCGATGGAATTGCA
AAGCTTGCGGATTTCGGTGTCGCTGGACAGTTGACTGATACAATGGCAAAAAGAAATACA
GTCATTGGAACGCCATTCTGGATGGCCCCTGAAGTTATCGAAGAAATCGGATATGACACA
AAAGCTGATATATGGTCACTGGGAATAACAGCTATCGAAATGGCAGAGGGACGGCCTCCT
TATTCAGATATTCATCCGATGCGAGCAATCTTCATGATTCCAACTAAACCACCACCTACG
TTTAAAAAACCTGAAGAATGGTCGTCCGAGTTCAATGATTTCATAAGAAGCTGTTTAATT
AAAAAGCCAGAAGAAAGAAAAACGGCACTCCGATTGTGTGAGgtacgtatggcttaaaaa
aaaagaaatttcccaataagatttatccaaaaaatattgaaattctcaaatattcgttat
atttgttaaaactgtgaaaaaaggtacatccacctaaaattcaaaatttcgacaactttt
ttttgtcgcagggactagaaattaattttcaatcatgtagtatttctttttttctattttt
taaagtttctagtcggtagtcagctaaacttgtgttctaatcggctgaaaatatgtccat
gtcagcaaaaaatgcgagaaaagcgataaactttcacgtttgactacaaaatgatgtcga
taaaagcttgaaatctataaaaacaaacaatccaaaaaacgctagaactcaacataattc
tctactcggtattcagaggcaacttcctgccacactttgacaacttatttagtgccacta
aaataagttgccaaattgctggcaggaagttgccgagttgttggcaggaagttgccacgt
agtggcagtaagtgggaaaaaacatctaaatgttggcaggaatgtgcaaaagtttggcag
aaagttgccaaaagttggcaggaagttgcctttaaatgccgaggagcgttctttcccact
tgctgccactacgtggcaactcatttaaaaactgccaagatatagacggggccaaatttt
catatttcctacaactactctcaaccgaaaatagaagatttcttataatttgacaacccc
ttcggagtaatttttaactttttacatagaattttctactatttttatacactttttttg
ttgttttctgtcatagtattactaatactgctgaaaaacaaatgatacacagtttgtgaa
cattgctcacttcatagatatttttcagCACACATTCATCAAAAATGCACCAGGTTGCGA
TATTATGCAGTTGATGATCCAGGATGCTCAAGAAAAAGCTATACTAGGACAAGCACCAAT
GgttgacaaaaaatttcattagaaaacgaaaatttgattgtttttcagGCAGCAAGCAG
CGGAAATGATGCAACGTTGCTAAGCGAGGGAATGTCCACTATGATTGACGGTGGAGAGTC
TACGTTAGTTCAACACAAAGgtattatgatagttttgaatatgtaatttgatcatatgtg
tcgtgggaaagtgcaaaactcacgcacctaatttgcactactttgtagctcgatatttag
tagtaacgtgaagcaggacacagggagaaattacaaaattacctagtgaaatagaactca
caatttcagACAACTATGTCACTGCTCAAAGTCTTCGAAGTCAAATGGAAAGCTTGAGAA
TTGGTGGAGAAATTCCTAAATCGGCATATAgttcgtttttcatgagcgttaaattgcat
tcaccgatatagttccag**GCTCGTCGAAAAATGGCAACTCACCGCGCGTTCAACCACCGG**
GTCACACCGCAAGCGCTTCAGACCCTTCAAAAAACCAACCGTTTGCTCAAGATGGAACCG
GTCCAAACTTTCAATTGGGCACCAGCGAGAGCAGTTACAAAGATGCTAGTTATAATATGA
gttagttatttgaaagaaaaccaattataaaatgatgatctatgagaaaaaaaagctagc
ataaaaaacgttattcagctcgaatgactttgacaaaaaaatgaaaaattattcaggcat
tttcacaaacctaaaattttattaaaattgaaatgtcataccccttattctagccatgca
tgaaatgttagctgattaagattttgcctgtaaattctaaaaaatgaaaaatgtcactac
taatgttttgtggaaaatttgtattgcataagttttgaaacttagaaagacatattttga
aatcttgaattgaatcacagttaaaaacacctactctttcagTGAACACGGAAGCCGAA
TACGAAAATAGATTTCAACGGGCAGTGGTCGATGGAGATTTCGAATTTgtaagtgtattt

bioRxiv preprint doi: https://doi.org/10.1101/2025.08.22.671798; this version posted August 29, 2025. The copyright holder for this preprint (which was not certified by peer review) is the author/funder, who has granted bioRxiv a license to display the preprint in perpetuity. It is made available under aCC-BY 4.0 International license.

cgtaattttctaataacgaaatagctgcatttagTTACGAAACATCACGCTAGACGAAC
TGATTCGAAGGAAAGAAAGTTTGGATTCGGAGATGGAAGAAGAAATACGAGAGTTGCAGA
GAAGgtaaaatcttttttttttaattggttgaatttgaggaaatttaattcaaacacctc
aaaaagccggaaattgagattttaacaaaaaatcctaagcgaattttctagctggattt
tttgtgagtttactgatttaaacatctaaaggacagttttatgaatcgcgaactactgct
atttataatttttaagaaatagtgatgctgaaattttgctcagaatttttttctttgatg
cttaaaattccattctcaaatttaggatatttgaagcttttggcatcgaatgttttcaaa
atgagccaacttttctctttttttcttacattttggagtcaagaaaattgatattaaaat
ccgcaacagtggcaattcacgattaaagtcaaatactaaaaaaagaacaaaatttcagAT
ACAAAACAAAAAGGCAACCAATACTGGATGTTATAGAAATCAAAAAACGACTTCAA**TGA**c
ggcttcgatacatgcttgtttaataataatttttttgtcaatctgcaaatatatgaaacc
tattaattaactagattgtattttaaatgtcatatttattctaaattttgttagatcaga
agcaagagcaaactgaacttgttgtgttttgcttagctttttcatgctaaaataaatctg
gtcaattttaatttttttgggaaggtttgttctgaaggcggatattttatcagtgactaa
caaggaacaatgggagtgacggatataatttttcgaataacggaaatagataaaatttca
cttacaattgaaaaagctgcaatgagtcagagcgatagatggttgatctcgatgagtcta
gtttttcttatttgtttgcctaccgcggagttcacgtagcgaccaattaatatttttagg
taaattttgcatggcatcctagtagaaacaaaaaaccacattctgagtgctttttgcatt
acgactgccgttttgaatcgtaagatttatggcttatgacggacgtcctttttagctttag
tctgttataattgtcatattgggtctctgctgccgttgggaagcagatatatttcgtcaa
ctactagccgagagggcgtttgcttttttttttttcaagaagaggcaaatatacacttcga
ggcgaatgtttctcaactgtcaggtaatatttgcgaaaccgaaatatatttaaggagtga
aggaagcgaagattgccatcgaacaaaagttcatccgctatcagcttatcaacaatgttc
gcacttattctttctttgtgtctttttcaaggtaaactattctttcagaattatagtatt
ttctgaaaaatgtttccaatttctgaaacagtttttttttgattgttataaaatattttt
aggaacactttcatatgtaattggaggcggtgtacaagcaaatccaagcggatgcaacgt
atgtggtgatagccctcagtggagtggatggacagaatggtcttcctgttctgccgcttt
tggaagtaggtcttctatttttttgaattttgctatagcagttttgcgaaaacctgacac
atcaatttaaagaaacattcagttttactggtcccgccacaatttcagtgatctttatga
aatgtaaaattctacagtgcacagagattcttggatttgaaatagaatttccaacacctg
gagtcacatttccgctatatgtacatcttcataataataatttcaatttcagcgccagtc
caaactcgcactagactttgtccatccggcaactgccaaggaggttctacaacagaatca
aaaccatgcgtcctttatgatccacaaccaactcaaccacaatggggagcttggggaggt
tggagtagttgcaggttagcaatattttgctttaatctgtttattattgttttgttttct
ttattgccaaactgataagcctaactttttctttttgcagtgccacctgcggcggtggtac
gatgacgagaagtcgtgtttgcaataacggatgctcaacctgccaatgtgtcggagctgc
tgccgagtcgcaagcttgcaatgctcaaccgtgctgcacctggactgctgtgagaacaaa
ttgagttcaacttctcacttaataacttttgtttcagtggtcttcctggtcggcgtgctc
agtaacctgcggatctggaggatcaatcaccagatctaggcaatgtagctgtggatcggg
agtaagtttaaaatttataaagctaaacgtttttattcaatataacatttataactgttt
gtttcagtgtactggaggctccgttgaacaagagccatgccctcaacaagctgcttgccc
gtgcacaacatgcaatcaaccaccaccaccatgcaatacttgtaatacccaaccggttgt
cattgtgactccagctccatgcacaacttgttaccaaccaccagcgtgctcaacctgcgg
gcatgcacaacctttctatgatccatacggaaatggaagaaagaagagaatgattacagt
atctggaaattcgacaagtgcttaactgtattgaaacttgtgtacatttatgcaatattt
tgcacattccgaagcaatataaataaagtattgagcaattattatttctatccactacag
cagaatcacagaaccactaaaaaaaacaaa

bioRxiv preprint doi: https://doi.org/10.1101/2025.08.22.671798; this version posted August 29, 2025. The copyright holder for this preprint (which was not certified by peer review) is the author/funder, who has granted bioRxiv a license to display the preprint in perpetuity. It is made available under aCC-BY 4.0 International license.

***cst-2* genomic seq annot:**

Lt gray: UTR
Yellow: alternating exons
Green: alternating exons
Blue: two-codon alternative splice in intron 8.
Purple: exon 9 sequence that is IDENTICAL with cst-1 exon 9.
Pink: start of next gene downstream, unrelated

>Unspliced + UTR + upstream + downstream (6994bp)
taattgatttttcaaaaatatatacacccataatatgaaaaagtattttcaactatcaat
aaacaccgagaaaagcggaatatcaaaatttttggcaatttgacaatcgataattttga
acaaataaaattcaaaaacaaacaaacaaacaatagttcattgtaataaatatttttggg
aagacatacaacgtttccctactaaatgtctgctattccagattgccgagtttgaaaata
ttcggtattttcgggaatcgccgaatcttgaattcatcagcaattcaatattttccgcat
tttaacaattgcctcccacccctaattcataaaaatttgatattccgcttttctcggtg
tttattgatagttgaaaatactttttcatattatgggtgtatatatttttgaaaaatcaa
ttaacagttctttaaagttaaatagattgaaattaaagaataacttggaaattgtttttg
aatttatgacatctctttgcttgaaaaaatggatgaaacatgctttataaaatcaactag
aataattgtccaaactgacaaaaagcaatcaactactatgcaccataacaaataaagtct
actgacaagtaaagatagtaaacatacgtgaatcaggtgatgactacccaacaactctca
taataaaacagtaccgcaaaacagcaccaggaaggacacagttagcaatacgatattccg
cattcaaacatcctgacgtgtgaagggtcaagtcttatgaaacgagcgcagtacacactt
cgcactctttcccgtccttttccccctttcgcctcgaccacacactgctcaaataacgta
gagtgagccgagggcattacgaatgctcgtcgaatgtagcgcctgcggtcggttatatta
acttctttttttttatcacggtacaacttatctgaaatagagtactgtttttgagttgtt
ctctggtctttgatagtttgatagtttcaatgggaatatttaattctcacactttcaaaa
aggatatttcaatcttaaatgaaatgctaacattgcgaaatagaattgaaaatcatgata
cttcaatctctttcatgaaaaataaggttcttctaaatttgtattgttttaaaatatgct
caggtctatctcaaagatttcataaattataaaatttattaattagtttttctcggcttt
ttcaaccattttaccaatgaaatcttaatctaaaaacgttggactttgtcggattacttt
tttttccaaaagacaattgccgtagtcaattattcattcttcgggccatcggggccacat
gggtggaaccccgcagacgtcttttcgctttcaaaacgacgtccaaatcgattcgcctgt
ctttctcttctcactcttctcgcctcccttcagaatatgaccatacatgcactgttgacg
ttctaattgaacaagcagagtgcatgtgcaaccaagagaacgcagagaaacatgcgacta
atgtgtcctcaaggataggttttatcttcttcttcttctttcttcttctttcttcgattt
tcttgccccatagttctcgtcggtcggacggacaacatctgctcttcatcttacttatat
ctcattgggtgttcattcctatactcacacaagatcttacaacactttttttataaatcct
tcaacttgtacacagttaaaggtggagtagcgcaagttggaaaattgtttaaaactactc
ttatggtgtcaaaatgactaaatattaaacaaataaaaatgctgaacagtttttgaaagg
acgactttcaaaaagtccaacaattggcaaaaactgagtttattttcaattttcaaaaaa
aaatcagataaaatttagaacatctttcagaaactttttgctaaaatatttgattattgt
ggtgccgcatgcatgtttttgagcaatttcagcactgacgttactccacttttaaatttc
agcaaagttaatttcattaatttcagcgtcctcccg**ATG**CCACCGTCTACAGACAGCTCG
CGGCGGAATTCGGAAGAGGGCTCAAGgtttgtcagacacaaaacaaaaaaaaagagttac
tcttccaaaataaacattctcttttgttttagCGATGGATTTAAATTGGATTCGTCGGCG
CTTAACAAACCACCAGAAGAAGTTTTCGACATTGTTGGAAAGCTGGGCGAAGGgtttgtt
atttcaattctatcggattgaaaaattccaaattttcagATCATATGGAAGCGTACATAA
GGCAATTCACAGAGAGTCAGGTCATGTGTTAGCCATCAAAAAAGTGCCAGTGGATACTGA
TCTTCAAGAAATTATCAAGGAAATCAGTATAATGCAACAATGTAAAAGTAAATATGTTGT

bioRxiv preprint doi: https://doi.org/10.1101/2025.08.22.671798; this version posted August 29, 2025. The copyright holder for this preprint (which was not certified by peer review) is the author/funder, who has granted bioRxiv a license to display the preprint in perpetuity. It is made available under aCC-BY 4.0 International license.

CAAATACTATGGATCGTATTTCAAACATTCGGATCTATGGgtgagattgtttagataaat
tttaagattttaatgctgtgcccaagtgggactaaataaaagaaattgaacaaaaattat
attcgttttgtgaattagagttaaaaacagtaaagttctgatttttccacagaaaaaaat
gtagcttaaagtggaaataacattcaaaagtattcattcaaatgcatgccaattgaaatt
caaagattttattccctacccctagtcgaaaatcaagaagaaaactggactaacaaaaaa
actaatttaaagATTGTGATGGAATACTGTGGAGCTGGGTCGATCTCCGATATAATGCGT
GCCAGGCGGAAACCGTTATCCGAACAAGAAATCAGTGCAGTTTTGCGCGATACTCTCAAG
GGATTGCAATATTTGCATGACTTGAAGAAGATTCATAGAGATATAAAGgttagaaactgt
tttttttgaaaaaaaaactgtattttcagGCTGGAAACATTCTACTCAATACCGATGGA
ATTGCAAAGCTTGCGGATTTCGGTGTCGCTGGACAGTTGACTGATACAATGGCAAAAAGA
AATACAGTCATTGGAACGCCATTCTGGATGGCCCCTGAAGTTATCGAAGAAATCGGATAT
GACACAAAAGCTGATATATGGTCACTGGGAATAACAGCTATCGAAATGGCAGAGGGACGG
CCTCCTTATTCAGATATTCATCCGATGCGAGCAATCTTCATGATTCCAACTAAACCACCA
CCTACGTTTAAAAAACCTGAAGAATGGTCGTCCGAGTTCAATGATTTCATAAGAAGCTGT
TTAATTAAAAAGCCAGAAGAAAGAAAAACGGCACTCCGATTGTGTGAGgtacgtatggct
taaaaaaaaagaaatttcccaataagatttatccaaaaaatattgaaattctcaaatatt
cgttatatttgttaaaactgtgaaaaaaggtacatccacctaaaattcaaaatttcgaca
actttttttgtcgcagggactagaaattaattttcaatcatgtagtatttctttttttct
atttttaaagtttctagtcggtagtcagctaaacttgtgttctaatcggctgaaaatat
gtccatgtcagcaaaaaatgcgagaaaagcgataaactttcacgtttgactacaaaatga
tgtcgataaaagcttgaaatctataaaaacaaacaatccaaaaaacgctagaactcaaca
taattctctactcggtattcagaggcaacttcctgccacactttgacaacttatttagtg
ccactaaaataagttgccaaattgctggcaggaagttgccgagttgttggcaggaagttg
ccacgtagtggcagtaagtgggaaaaaacatctaaatgttggcaggaatgtgcaaaagtt
tggcagaaagttgccaaaagttggcaggaagttgcctttaaatgccgaggagcgttcttt
cccacttgctgccactacgtggcaactcatttaaaaactgccaagatatagacggggcca
aattttcatatttcctacaactactctcaaccgaaaatagaagatttcttataatttgac
aaccccttcggagtaatttttaactttttacatagaattttctactatttttatacact
tttttgttgttttctgtcatagtattactaatactgctgaaaaacaaatgatacacagtt
tgtgaacattgctcacttcatagatatttttcagCACACATTCATCAAAAATGCACCAGG
TTGCGATATTATGCAGTTGATGATCCAGGATGCTCAAGAAAAAGCTATACTAGGACAAGC
ACCAATGgttgacaaaaaatttcattagaaaacgaaaatttgattgtttttttcagGCAGC
AAGCAGCGGAAATGATGCAACGTTGCTAAGCGAGGGAATGTCCACTATGATTGACGGTGG
AGAGTCTACGTTAGTTCAACACAAAGgtattatgatagttttgaatatgtaatttgatca
tatgtgtcgtgggaaagtgcaaaactcacgcacctaatttgcactactttgtagctcgat
atttagtagtaacgtgaagcaggacacagggagaaattacaaaattacctagtgaaatag
aactcacaatttcagACAACTATGTCACTGCTCAAAGTCTTCGAAGTCAAATGGAAAGCT
TGAGAATTGGTGGAGAAATTCCTAAATCGGCATATAgttcgtttttttcatgagcgttaaa
ttgcattcaccgatatagttccag**GCTCGTCGAAAAATGGCAACTCACCGCGCGTTCAAC**
CACCGGGTCACACCGCAAGCGCTTCAGACCCTTCAAAAAACCAACCGTTTGCTCAAGATG
GAACCGGTCCAAACTTTCATTCCACTATCTCAACACTTTGTTTTGGTTTTTATAATT**TAA**a
agcctagctaatatcctaatctgtttacctgatataatagttttcaaatttaccttctgg
aaaacttatatattactatactgtcgaaacggatgccgcacatccctttgctgtaaaaat
tgccgagttcctaactctttgtccgcaaaactcacaaatagtctatgagataatgcatgt
tctcttctgaacctttcaaatcaactatagctggtaagtactctttcaattagttcagct
tcttttcttttctcaccaactactatctcgactccgacctatttagatcggaaccaattc
tctcccgatcacttcaacatatgaaaaagacgaatttgtgagttagagagtatactcgaa
atagtaacattgatttcccccatttttcgactttcttttcttcgctatcgtgtgtactta
tcgcatgtaacatttgctgattagctgactgaccacggcttcaagtagcaaatttatttc

bioRxiv preprint doi: https://doi.org/10.1101/2025.08.22.671798; this version posted August 29, 2025. The copyright holder for this preprint (which was not certified by peer review) is the author/funder, who has granted bioRxiv a license to display the preprint in perpetuity. It is made available under aCC-BY 4.0 International license.

```
tagcacgtgaggttgcatagatgagtaccttcgttagctttgctttcaggaactcaaaat
tgaaaagagaaacatgtggtgaagcagcggtaggtgaaactctttttctaagctattgca
agattttctcaagatcacacgcctagtatgcagggtgagaatgccggatgcattgaaaat
aaatgcaacaagttgcagctatgattagcttttttttaatgattcgactgctttcagaatg
gctttatattgcaccagctctttgagggatctttgaataatttttttcaggttgaaaagc
gaaagatgagacgtttatccgagatgatatctgattgggaacgaccgtcaagaagttcga
tcagtaacatgtatctttatgcgagaagagaaagcgtctacattgcaaggaagaagttgg
ccggtctgaaagtaataatttgagctttaaaaacaaacacactataaagactgaaaataa
tccgtttttaatacaaaactgtttaaaaaaatttaaaaaagattcatacttgttgttgtt
ctagaatgttcatggttcaatttagattagctcaagtatgattttaaatatttttttgtta
tatccagattgaaaatacgtagaatattcaaagaaagacattgaaatttgaattacctgc
aaaatacgcatgtttcagccacttttggtaactcaacgctaaattttcataaaaatttttc
catatcctttgaaacatccggaatctaaaaaaaaacaagaaaaaaacggaaaatattgga
actataccaaaataaaattaccgaattttcttcaactgtccgattttccaggactgggcg
ctggacctgttagcaaaactgaaatccaagcagggaatggcgattcgtcgagagactcgt
gcaaccaagttggttgcaactgtaatgggtaactgaaactattctttgacattaaaacta
aaccaaatacattgcagtggtttttctcgtctgctggcttccgtttttcacattgaacat
gatcaaaatttacaaattgatattcaacgtttggtcagcggacctcgaaatttggttcca
ttggtttaccgcactggggtatttgaattcgtcactcaactttttcatttactcaacaat
caatccagtaagcatttttatatcaaatttgatagtaattatagaaataattataataca
taattggctatataataaaaaagtttagtcgaaaaagattatatttttaatttttcaagt
tcaatttgcgttgtataaaacattaattcatggaattgagacaaggtaaaaccgacaacg
ttttccaaaattttgacatgtctgtattccctctataggcattgcgccagcttaactcaa
tttgacaagaaatagtaaacccgcttagcaaactaaatatactgaaaactgtactgtata
atccattcaacacctcctttttcagaaattccgtcattcattccgccgcttacttggct
tccgtcgatcttctcgacgtcagagagaaaaatcgtggatgttaccgccacgagatagca
gcaggtgagcctcatttattcgtccatagcttttaaatgtttcataacagaggtcctgca
aaatgtggatgcggtttccataaaatcgtgaaaa
```

Huynh Fig. S5

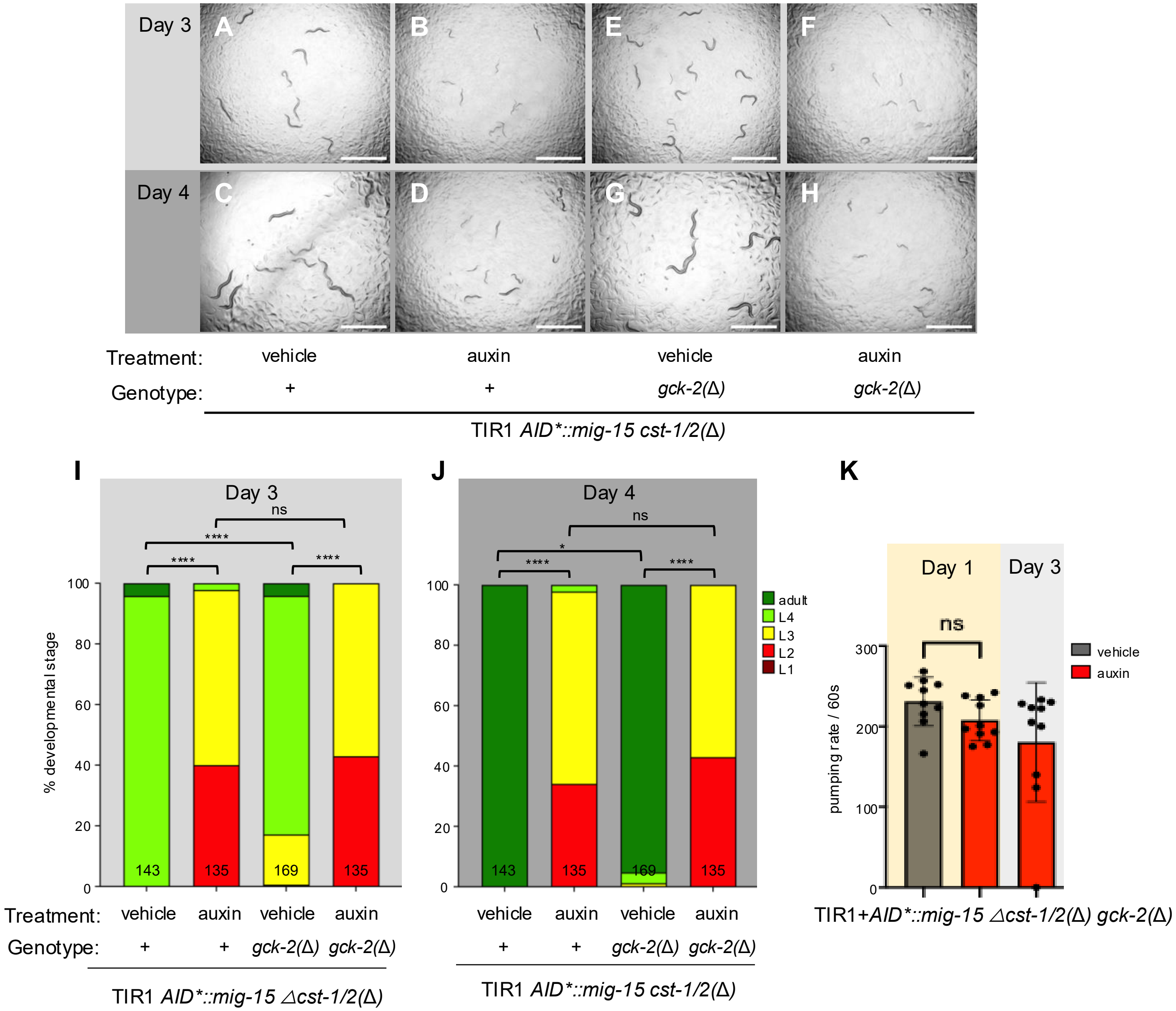

Day 3
Day 4
A
B
E
F
C
D
G
H
Treatment: vehicle auxin vehicle auxin
Genotype: + + gck-2(Δ) gck-2(Δ)
TIR1 AID*::mig-15 cst-1/2(Δ)
I
Day 3
ns
****
****
****
% developmental stage
100
80
60
40
20
0
143
135
169
135
Treatment: vehicle auxin vehicle auxin
Genotype: + + gck-2(Δ) gck-2(Δ)
TIR1 AID*::mig-15 △cst-1/2(Δ)
J
Day 4
ns
*
****
****
143
135
169
135
adult
L4
L3
L2
L1
Treatment: vehicle auxin vehicle auxin
Genotype: + + gck-2(Δ) gck-2(Δ)
TIR1 AID*::mig-15 cst-1/2(Δ)
K
Day 1
Day 3
ns
300
200
100
0
pumping rate / 60s
vehicle
auxin
TIR1+AID*::mig-15 △cst-1/2(Δ) gck-2(Δ)

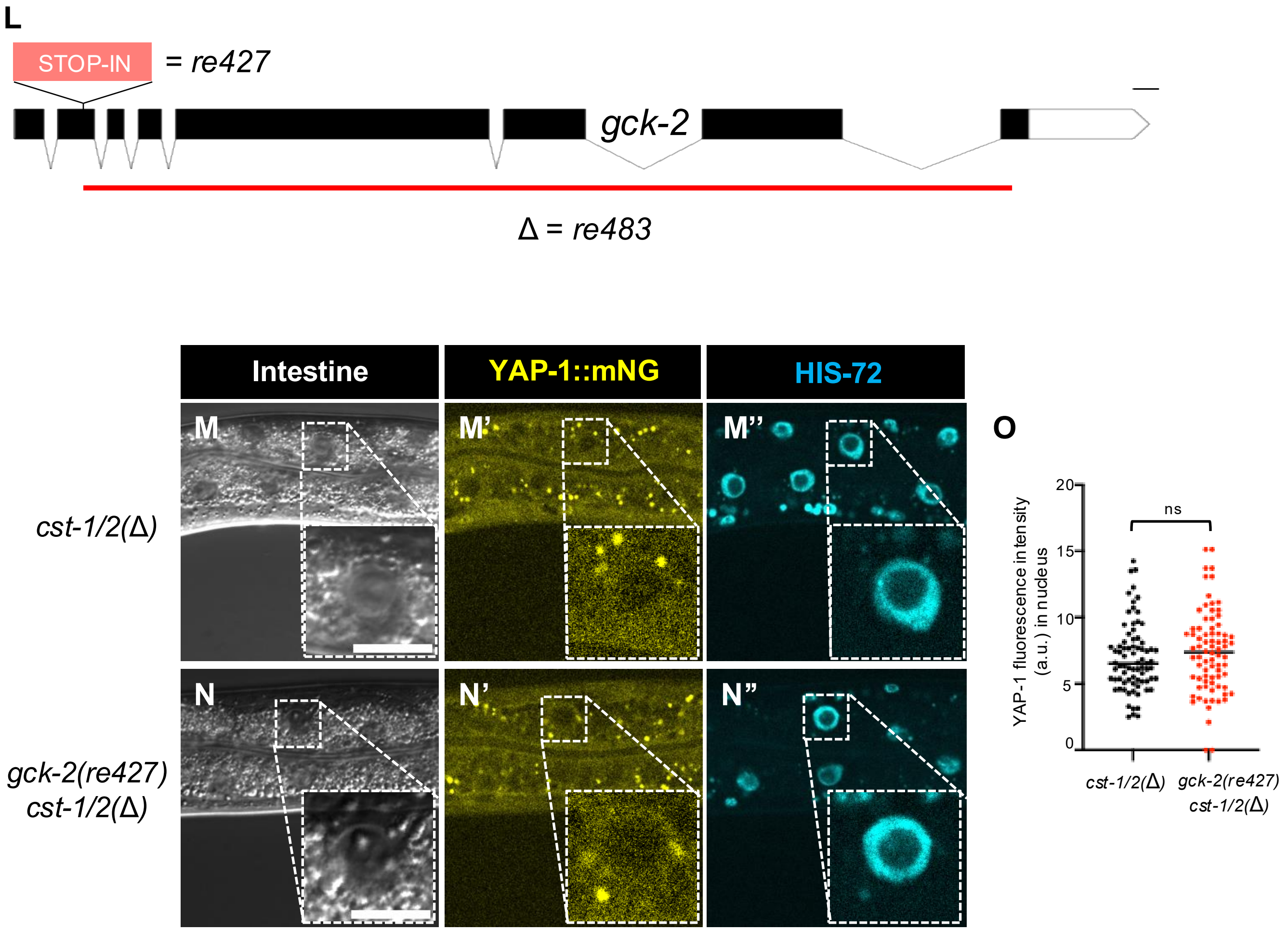
L
STOP-IN
= re427
gck-2
Δ = re483
Intestine
YAP-1::mNG
HIS-72
M
M'
M''
cst-1/2(Δ)
N
N'
N''
gck-2(re427)
cst-1/2(Δ)
O
YAP-1 fluorescence intensity (a.u.) in nucleus
20
15
10
5
0
ns
cst-1/2(Δ)
gck-2(re427) cst-1/2(Δ)

Huynh Fig. S6

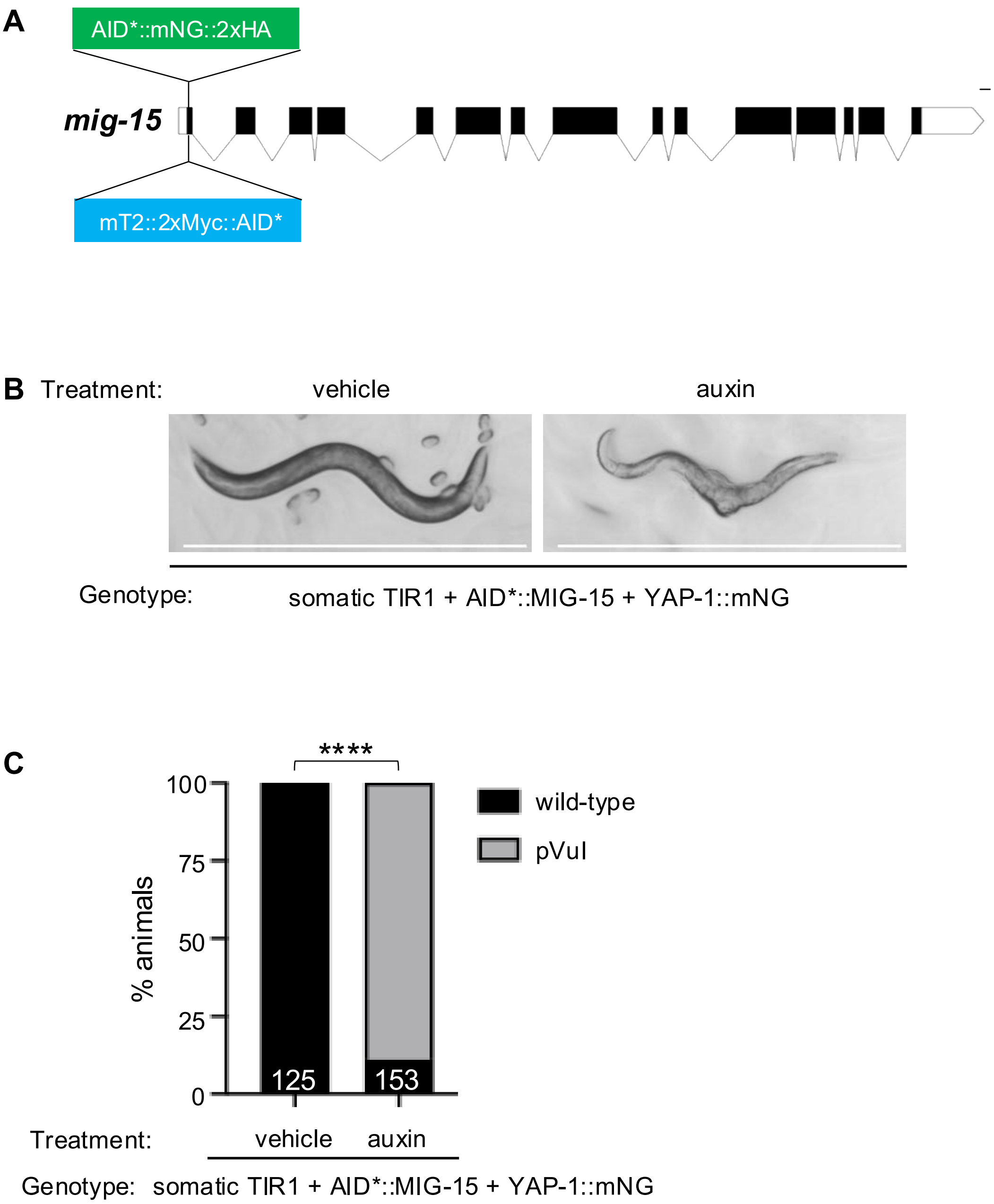
A
AID*::mNG::2xHA
mig-15
mT2::2xMyc::AID*
B
Treatment:
vehicle
auxin
Genotype:
somatic TIR1 + AID*::MIG-15 + YAP-1::mNG
C
****
100
75
50
25
0
% animals
125
153
wild-type
pVul
Treatment:
vehicle
auxin
Genotype:
somatic TIR1 + AID*::MIG-15 + YAP-1::mNG

bioRxiv preprint doi: https://doi.org/10.1101/2025.08.22.671798; this version posted August 29, 2025. The copyright holder for this preprint (which was not certified by peer review) is the author/funder, who has granted bioRxiv a license to display the preprint in perpetuity. It is made available under aCC-BY 4.0 International license.

SUPPLEMENTARY TABLES

Table S1: Strain

Strain	Genotype
CA1200	*ieSi57[eft-3p>TIR1::mRuby + Cbr-unc-119(+)]* II; *unc-119(ed3)* III
DV3709	*ieSi57[eft-3p>TIR1::mRuby + Cbr-unc-119(+)]* II; *unc-119(ed3)* III; *mig-15(re264[AID*::mNG::2xHA::mig-15])* X
DV3948	*ieSi57[eft-3p>TIR1::mRuby + Cbr-unc-119(+)]* II; *unc-119(ed3)* III; *mig-15(re264[AID*::mNG::2xHA::mig-15]) cst-1/2(reDf1[re484])* X
DV3969	*ieSi57[eft-3p>TIR1::mRuby + Cbr-unc-119(+)]* II; *unc-119(ed3)* III; *gck-2(re483)* V; *mig-15(re264[AID*::mNG::2xHA::mig-15]) cst-1/2(reDf1[re484])* X
DV3611	*yap-1(re269[yap-1::mNG::2xflag])* X
DV4205	*ieSi57[eft-3p>TIR1::mRuby + Cbr-unc-119(+)]* II; *unc-119(ed3)* III; *yap-1(re269[yap-1::mNG::2xflag])* X
DV4130	*rrf-3(re390[STOP-IN*])* II; *his-72 (erb77[his-72::linker::mTurquoise2])* III, *yap-1(re269[yap-1::mNG::2xflag]* X
DV4186	*his-72(erb77[his-72::linker::mTurquoise2])* III, *yap-1(re269[yap-1::mNG::2xflag] cst-1/2(reDf4[re452])* X
DV4217	*wts-1(tm4081)/tmC18[dpy-5(tmls1236[myo-2p>mCherry])]* I
DV4214	*wts-1(re419[mTurqoise2::2xMyc::AID*::wts-1])* I; *ieSi57[eft-3p>TIR1::mRuby + Cbr-unc-119(+)]* II; *yap-1(re269[yap-1::mNG::2xflag]* X

bioRxiv preprint doi: https://doi.org/10.1101/2025.08.22.671798; this version posted August 29, 2025. The copyright holder for this preprint (which was not certified by peer review) is the author/funder, who has granted bioRxiv a license to display the preprint in perpetuity. It is made available under aCC-BY 4.0 International license.

DV4288	*his-72(erb77[his-72::linker::mTurquoise2])* III; *gck-2(re427[STOP-IN*])* V; *yap-1(re269[yap-1::mNG::2xflag]) cst-1/2(reDf4[re452])* X
DV4290	*wts-1(re419[mTurqoise2::2xMyc::AID*::wts-1])* I; *ieSi57[eft-3p>TIR1::mRuby* + *Cbr-unc-119(+)]* II; *unc-119(ed3)* III
DV4292	*wts-1(ok753)/tmC18[dpy-5(tmIs1236[myo-2p>mCherry])]* I
DV4278	*wts-1(tm4081)* I; *yap-1(re269[yap-1::mNG::2xflag])* X
DV4297	*wts-1(re436[STOP-IN*])/tmC18[dpy-5(tmIs1200[myo-2p>Venus])* I
DV4296	*ieSi57[eft-3p>TIR1::mRuby* + *Cbr-unc-119(+)]* II; *unc-119(ed3)* III; *mig-15(re435[mTurquoise2::2xmyc::AID*::mig-15) yap-1(re269[yap-1::mNG::2xflag])* X
DV4337	*wts-1(re419[mTurqoise2::2xmyc::AID*::wts-1])* I; *reSi2[col-10p>TIR1::F2A::mTaqBFP2::AID::NLS]* II
DV4338	*wts-1(re419[mTurqoise2::2xmyc::AID*::wts-1])* I; *reSi12[ges-1p>TIR1::F2A::mTaqBFP2::AID*::NLS]* II
DV4339	*wts-1(re419[mTurqoise2::2xmyc::AID*::wts-1])* I; *reSi2[col-10p>TIR1::F2A::mTaqBFP2::AID*::]* II; *yap-1(re269[yap-1::mNG::2xflag])* X
DV4340	*wts-1(re419[mTurqoise2::2xmyc::AID*::wts-1])* I; *reSi12[ges-1p>TIR1::F2A::mTaqBFP2::AID*::NLS]* II; *yap-1(re269[yap-1::mNG::2xflag])* X
DV4341	*wts-1(re419[mTurqoise2::2xmyc::AID*::wts-1])* I; *ieSi68[sun-1p>TIR1::mRuby* + *Cbr-unc-119(+)]* II

bioRxiv preprint doi: https://doi.org/10.1101/2025.08.22.671798; this version posted August 29, 2025. The copyright holder for this preprint (which was not certified by peer review) is the author/funder, who has granted bioRxiv a license to display the preprint in perpetuity. It is made available under aCC-BY 4.0 International license.

DV4384	*ieSi57[eft-3p>TIR1::mRuby + Cbr-unc-119(+)]* II*; unc-119(ed3)* III; *mig-15(re435[mTurquoise2::2xmyc::AID*::mig-15) yap-1(re269[yap-1::mNG::2xflag]) cst-1/2(re479)* X

Table S2: Oligonucleotide

**Name**	**Oligonucleotide sequence**	**Use**
LH12	GATATTGCCGAGCACACATGG	genotyping triplex *rrf-3(re390)*
DJR881-RV1	GCTTATCACTTAGTCACCTCTGCTC	genotyping triplex *rrf-3(re390)*
LH13	GATGTGAAACTTGATGCGAACTCG	genotyping triplex *rrf-3(re390)*
LH11 repair *rrf-3*	AATTTCAGTCTAAAGTTGACGAGGTAAACTGGATCAGGGAAGTTTG TCCAGAGCAGAGGTGACTAAGTGATAAGCTAGCAGGTGGGGATG GTGCCACGAGCTGCGTACGAAGATAAA	repair template for *rrf-3(re390)*
LH60	CTTACTTTCTATTCAAGGTTTTATTTCAAACGTAGGACACC	genotyping triplex *wts-1(tm4081)*
LH61	TGCTGTAGAAGTCGTTCAACATGTTGTTCC	genotyping triplex *wts-1(tm4081)*
LH62	TGTCTTCGCAGACACCCATAATCAGC	genotyping *triplex wts-1(tm4081)*

bioRxiv preprint doi: https://doi.org/10.1101/2025.08.22.671798; this version posted August 29, 2025. The copyright holder for this preprint (which was not certified by peer review) is the author/funder, who has granted bioRxiv a license to display the preprint in perpetuity. It is made available under aCC-BY 4.0 International license.

LH200	AAAGCGGATGTCATAATGAAACAACAGGTAGG	genotyping triplex *wts-1(ok753)*
LH201	GTGAAAGCCGAACGCGATATTCTGG	genotyping triplex *wts-1(ok753)*
LH202	AGCTGCAACTTCAGGAGGAAGC	genotyping triplex *wts-1(ok753)*
LH131	CGGGAAGCAAAGAGACCACATAACG	genotyping triplex *wts-1(re436[STOP IN])* and *wts-1(re419[mTurqoise2::2xmyc::AID*::wts-1])*
LH133	AACCAAAACCTACTGAATTGTGATGGGC	genotyping triplex *wts-1(re436[STOP IN])* and *wts-1(re419[mTurqoise2::2xmyc::AID*::wts-1])*
DJR881-RV1	GCTTATCACTTAGTCACCTCTGCTC	genotyping triplex *wts-1(re436[STOP IN])*
LH134 repair wts-1 stop-in	GTGTTCGGCAGGAGGCCCTCCTTTGCATTGTATTCCCGGGAAGTTTGTCCAGAGCAGAGGTGACTAAGTGATAAGCTAGCTGCGACCTGCTGCCCCTGGTACTACTCCAAAT	repair template for *wts-1(re436[STOP IN])*

bioRxiv preprint doi: https://doi.org/10.1101/2025.08.22.671798; this version posted August 29, 2025. The copyright holder for this preprint (which was not certified by peer review) is the author/funder, who has granted bioRxiv a license to display the preprint in perpetuity. It is made available under aCC-BY 4.0 International license.

LH132 RV1_wts-1-AID	CGGTAAAGAGTTCTTCTCCTTTGGAGACC	genotyping triplex *wts-1(re419[mTurqoise2::2xmyc::AID*::wts-1])*
LH100 FW_repair mTurq2-wts-1	GGAGGCCCTCCTTTGCATTGTATTCCCCTGGAATGGTCTCTAAGGGAGAAGAACTCTTTACC	primer for amplifying fragment #1 repair template for *wts-1(re419[mTurqoise2::2xmyc::AID*::wts-1])*
LH101 RV_repair mTurq2-wts-1	ATCAGAAGATGCACCATTTGGAGTAGTACCTGGAGCTGCAGGTCGCATacCtGCgCCgCTTGCTCCtGATCCaGCaCCCTTCACG	primer for amplifying fragment #2 repair template for *wts-1(re419[mTurqoise2::2xmyc::AID*::wts-1])*
CB003 Deletion Repair Template	CAAAACAAAGTGTTGAGATAGTGGATGAAAGTTTGCAGTGGCAATTCACGATTAAAGTCAAATACTAAAA	repair template for *cst-1/2(reDf1), cst-1/2(reDf4) and cst-1/2(re479)*
CB004 CST FW	GGCATCCGTTTCGACAGTATAG	genotyping triplex *cst-1/2(reDf1), cst-1/2(reDf4) and cst-1/2(re479)*

bioRxiv preprint doi: https://doi.org/10.1101/2025.08.22.671798; this version posted August 29, 2025. The copyright holder for this preprint (which was not certified by peer review) is the author/funder, who has granted bioRxiv a license to display the preprint in perpetuity. It is made available under aCC-BY 4.0 International license.

CB005<br>CST RV-1	GCCACTGCAAACTTTCATCC	genotyping triplex *cst-1/2(reDf1)*, *cst-1/2(reDf4)* *and cst-1/2(re479)*
CB006<br>CST RV-2	TATGTGTCGTGGGAAAGTGC	genotyping triplex *cst-1/2(reDf1)*, *cst-1/2(reDf4)* *and cst-1/2(re479)*
RF21	TTAGCAACGATAACAATCGAGGAGTGGGATCAATGGTGCACCATT<br>ATCAAGGAGCATCGGGAGCCTCAG	primer for amplifying fragment #1 repair template for YAP-1 tag
RF22	AGAGAAAGAGAGTGGTTATTCTGCTGATTAGACATTTACGAGGCTC<br>CACGCTTGTCG	primer for amplifying fragment #2 repair template for YAP-1 tag
RF26 FW	CACACGAATTCGACCAGTATCTACA	genotyping *yap-1(re269[yap-1::mNG::2xflag])*
RF27 RV	CATCGACCATAGCGGCTTG	genotyping *yap-1(re269[yap-1::mNG::2xflag])*

bioRxiv preprint doi: https://doi.org/10.1101/2025.08.22.671798; this version posted August 29, 2025. The copyright holder for this preprint (which was not certified by peer review) is the author/funder, who has granted bioRxiv a license to display the preprint in perpetuity. It is made available under aCC-BY 4.0 International license.

RF28 RV	GTATTGGAAACGGGGTTGAGC	genotyping *yap-1(re269[yap-1::mNG::2xflag])*
LH004v2 GCK-2 Repair Template	GTGATTCACTGGCAGCTGTCAAAGTAGTCAAACTCGATCGCAACCAATGTCACCCCGAAACTCTGGCGATAC	repair template for *gck-2(re483)*
LH140 GCK-2 STOP-IN Repair Template	TGATTCACTGGCAGCTGTCAAAGTAGTCAAACTCGGGGAAGTTTGTCCAGAGCAGAGGTGACTAAGTGATAAGCTAGCAGGCGGGCGACAATTTTGCAGTAATCCAGCAGGAGATT	repair template for *gck-2(re427[STOP IN)*
LH80 GCK-2 FW	GGGTAATGAGTGCCGATGTAATCAAACG	genotyping triplex *gck-2(re483)*
LH81 GCK-2 RV-1	GAAGGCGATTTGAAGTTCCGAAAGTGG	genotyping triplex *gck-2(re483)*
LH83 GCK-2 RV-2	AGTTTCGGGGTGACATTGGTTGC	genotyping triplex *gck-2(re483)*
LH141	GGTAATGAGTGCCGATGTAATCAAACG	genotyping triplex *gck-2(re427[STOP IN)*
DJR881 stop-in RV1	GCTTATCACTTAGTCACCTCTGCTC	genotyping triplex *gck-2(re427[STOP IN)*
LH143	GCCGCAGTACTCCATAACAATCC	genotyping triplex *gck-2(re427[STOP IN)*

bioRxiv preprint doi: https://doi.org/10.1101/2025.08.22.671798; this version posted August 29, 2025. The copyright holder for this preprint (which was not certified by peer review) is the author/funder, who has granted bioRxiv a license to display the preprint in perpetuity. It is made available under aCC-BY 4.0 International license.

LH148_FW primer of AID-mig-15 repair template	TTGCTCCAAGCCGCTCACAGCACCCAAAACCATGTCAATGGTCTCCAAAGGAGAAGAACTC	primer for amplifying fragment #1 repair template for *mig-15(re435[mTurquoise2::2xmyc::AID*::mig-15])*
LH149_RV primer of AID-mig-15 repair template	AGAATTCAAATCAATCTCGTCGAGTCCTGATGACGACATACCTGCGCCGCTTGCTCCTGATCCAGCACCCTTCACG	primer for amplifying fragment #2 repair template for *mig-15(re435[mTurquoise2::2xmyc::AID*::mig-15])*
RF15_FW_AID-MIG-15	CATTGTGTGGTTTAAGTGTCGGC	genotyping triplex *mig-15(re435)*
LH151_RV1_AID-MIG-15	GTGCAAATGAATTTAAGGGTGAGTTTTCC	genotyping triplex *mig-15(re435)*
LH152_RV2_AID-MIG-15	CGTGTTGTTTGGTGTAGTTTCGC	genotyping triplex *mig-15(re435)*
*dpy-10(cn64*gf*)* ssODN repair template	CACTTGAACTTCAATACGGCAAGATGAGAATGACTGGAAACCGTACCGCATGCGGTGCCTATGGTAGCGGAGCTTCACATGGCTTCAGACCAACAGCCTAT	ssODN repair template for *dpy-10(cn64*gf*)*

bioRxiv preprint doi: https://doi.org/10.1101/2025.08.22.671798; this version posted August 29, 2025. The copyright holder for this preprint (which was not certified by peer review) is the author/funder, who has granted bioRxiv a license to display the preprint in perpetuity. It is made available under aCC-BY 4.0 International license.

Table S3: CRISPR guide RNA

RNA	Sequence	Use
LH10 rrf-3 crRNA-1	CGAGGUAAACUGGAUCAAGGGUUUUAGAGC UAUGCUGUUUUG	crRNA for *rrf-3(re390)*
LH130 crRNA rev wts-1	GGCAGCAGGUCGCAUUCCAGGUUUUAGAGC UAUGCUGUUUUG	crRNA for *wts-1(re436)* and *wts-1(re419)*
CB001 cst-2 crRNA	UAGUGGAUGAAAGUUUGGACGUUUUAGAGC UAUGCUGUUUUG	crRNA#1 for *cst-1/2(reDf1), cst-1/2(reDf4)* and *cst-1/2(re479)*
CB002 cst-1 crRNA	AUCGUGAAUUGCCACUGUUGGUUUUAGAGC UAUGCUGUUUUG	crRNA#2 for *cst-1/2(reDf1), cst-1/2(reDf4) and cst-1/2(re479)*
RF20	CAATCGAGGAGTGGGATCAAGUUUUAGAGC UAUGCUGUUUUG	crRNA for C term tag *yap-1(re269[yap-1::mNG::2xflag])*

bioRxiv preprint doi: https://doi.org/10.1101/2025.08.22.671798; this version posted August 29, 2025. The copyright holder for this preprint (which was not certified by peer review) is the author/funder, who has granted bioRxiv a license to display the preprint in perpetuity. It is made available under aCC-BY 4.0 International license.

LH005 GCK-2 crRNA-1	CAAAGUAGUCAAACUCGAGGGUUUUAGAGC UAUGCUGUUUUG	crRNA for *gck-2(re483) and gck-2(re427)*
LH006 GCK-2 crRNA-2	GUGACAUUGGUUGCGAUCGGGUUUUAGAG CUAUGCUGUUUUG	crRNA for *gck-2(re483)*
crRNA guide for mig-15 5' end	GTCGAGTCCTGATGACGACAGUUUUAGAGC UAUGCUGUUUUG	crRNA for tag *mig-15(re435[mTurquoise2::2xmyc::AID*::mig-15])*
dpy-10 crRNA	GCUACCAUAGGCACCACGAGGUUUUAGAGC UAUGCUGUUUUG	crRNA for *dpy-10*
tracrRNA	AACAGCAUAGCAAGUUAAAAUAAGGCUAGU CCGUUAUCAACUUGAAAAAGUGGCACCGAG UCGGUGCUUUUUUU	universal

bioRxiv preprint doi: https://doi.org/10.1101/2025.08.22.671798; this version posted August 29, 2025. The copyright holder for this preprint (which was not certified by peer review) is the author/funder, who has granted bioRxiv a license to display the preprint in perpetuity. It is made available under aCC-BY 4.0 International license.

Table S4: Plasmids

**Plasmid**	**Use**	**Note**
pNR pBS mNG::2xFLAG	tag *yap-1(re269[yap-1::mNG::2xflag])*	sequence verified
pYW2 pBS mTurq2::2xMYC::AID*	tag *wts-1(re419[mTurqoise2::2xmyc::AID*::wts-1])* and *mig-15(re435[mTurquoise2::2xmyc::AID*::mig-15])*	sequence verified
pREW2	*luciferase*(*RNAi*)	sequence verified
I-5K13	*wts-1(RNAi)* (T20F10.1)	sequence verified
X-5M19	*yap-1(RNAi)* RNAi (F13E6.4)	sequence verified
X-11E16	*egl-44(RNAi)* (F28B12.2a)	sequence verified
I-1K04	*pop-1(RNAi).* (W10C8.2)	sequence verified
